# Pulmonary Effects of Neonatal Hydrocortisone Treatment in Ventilator-Dependent Preterm Infants

**DOI:** 10.1155/2011/783893

**Published:** 2011-12-20

**Authors:** Sandra E. A. de Jong, Floris Groenendaal, Frank van Bel, Karin J. Rademaker

**Affiliations:** Department of Neonatology, University Medical Center Utrecht/Wilhelmina Children's Hospital, P.O. Box 85090, 3508 AB Utrecht, The Netherlands

## Abstract

*Background/Objective*. Hydrocortisone, administered to ventilated preterm neonates to facilitate extubation, has no adverse long-term effects, but short-term pulmonary effects have not been described previously. In the present study, we analyzed effects of hydrocortisone on ventilator settings and FiO_2_ in ventilator-dependent preterm infants. *Patients and Methods*. Fifty-five preterm children were included in this retrospective cohort study. Hydrocortisone was administered at a postnatal age of > 7 days to treat chronic lung disease (CLD). Ventilator settings before and after hydrocortisone administration were recorded as well as FiO_2_ at 36 weeks' gestational age. Presence of cerebral palsy was assessed at a mean corrected age of 24.1 months. *Results*. Hydrocortisone administered at a median postnatal age of 14 days significantly reduced FiO_2_ from a median of 0.39 to 0.30, mean airway pressure (MAP) from a median of 10.0 cm H_2_O to 7.6 cm H_2_O, and PaCO_2_ from a median of 53.5 mmHg to 47 mmHg. Extubation was achieved in all patients. CLD at 36 weeks was present in 11 of the 52 patients (21.1%). None developed cerebral palsy. *Conclusions*. Hydrocortisone was effective in reducing the FiO_2_, MAP, and PaCO_2_ and facilitated extubation. Hydrocortisone was not associated with cerebral palsy.

## 1. Introduction

Bronchopulmonary dysplasia (BPD) or chronic lung disease (CLD) was first described in 1967 as a severe form of lung disease in premature infants with severe respiratory distress syndrome, who received prolonged mechanical ventilation and high concentrations of oxygen [1]. Improved mechanically ventilatory strategies, and the introduction of antenatal glucocorticosteroids and postnatal surfactant treatment, have greatly reduced postnatal lung injury and increased survival of premature infants, resulting in a reported incidence of CLD of 8%–35% [2, 3]. 

Still, CLD remains a significant problem in neonatal intensive care because of the significant cause of mortality, morbidity, and prolonged hospitalization [3]. Since inflammation plays an important role in the pathogenesis of CLD [3, 4], glucocorticosteroids, mainly dexamethasone, have been widely used to reduce CLD. Numerous studies show that dexamethasone improves short-term respiratory function, leading to a reduction in supplementary oxygen requirements and earlier extubation [5–7].

Despite its positive effect on neonatal lung function, dexamethasone therapy exerts several acute adverse effects, such as hyperglycemia, hypertension, and left ventricular hypertrophy. Concerns about the long-term benefits of this steroid in ventilator-dependent infants arose in the 1970s when it became clear that there was an increased risk of cerebral palsy after postnatal dexamethasone exposure [8]. In recent years, scientific evidence has accumulated on the long-term negative effects of neonatal dexamethasone treatment [9–11].

In contrast to other neonatal intensive care units (NICUs) in the Netherlands, the Wilhelmina Children's Hospital has historically always used the corticosteroid hydrocortisone instead of the more potent dexamethasone to treat ventilator-dependent preterm infants at risk for CLD because of fewer adverse effects and the apparently similar clinical benefits [12, 13]. However, until now no studies have described the short-term effects of hydrocortisone treatment on respiratory function.

The purpose of this retrospective study is to analyze the immediate effect of hydrocortisone therapy on mechanical ventilation settings in ventilator-dependent preterm infants. 

## 2. Patients and Methods

The study population presented in this retrospective cohort study consists of patients who were born after a gestational age of ≤32 weeks and/or a birth weight of ≤1500 grams between January 1, 2003 and December 31, 2007. These patients were consecutively admitted soon after birth over a period of five years to the Neonatal Intensive Care Unit of the Wilhelmina Children's Hospital, a tertiary referral centre.

One hundred and twenty-seven children born after a pregnancy of ≤32 weeks and/or with a birth weight of ≤1500 g had been treated postnatally with corticosteroids for several reasons.

Nine patients received besides hydrocortisone also dexamethasone (two during part of their treatment in another hospital and seven prior to extubation) and were excluded to avoid contamination of hydrocortisone treatment effects.

One hundred and eighteen infants born after a pregnancy of ≤32 weeks and/or with a birth weight of ≤1500 g received hydrocortisone treatment. Two patients received hydrocortisone as a replacement therapy for their congenital adrenal hyperplasia. Fifty-three patients received hydrocortisone to treat hypotension.

Sixty-eight patients were treated with hydrocortisone to facilitate extubation. In one case, the attending neonatological staff decided to stop treatment after one day of hydrocortisone treatment because of severe respiratory insufficiency and the poor prognosis based on pulmonary hypoplasia as a result of PPROM.

In twelve children treated with nasal continuous positive airway pressure (nCPAP) after having been ventilated, hydrocortisone was administered in order to avoid re-intubation.

These thirteen children were excluded.

Four infants in whom hydrocortisone treatment was started while being ventilated died in neonatal period on day 13, 18, 22, and 63; one of bullous emphysema and three of severe chronic lung injury with in one case in combination with a metabolic disorder of unspecified origin. These four children were not excluded.

Fifty-five out of the 118 children (46.6%) received hydrocortisone treatment for prolonged ventilator dependency.

### 2.1. Eligibility Criteria

Infants were eligible for the study if they met the following criteria: (1) gestational age ≤32 weeks and/or birth weight ≤1500 g, (2) ventilator dependency based on signs of chronic lung disease, (3) hydrocortisone treatment, and (4) availability of data as defined in the study protocol. Infants who were treated with dexamethasone, for example, after referral from other NICUs, were excluded as well as infants who were not ventilator dependent but had CPAP for respiratory support.

### 2.2. Study Protocol

#### 2.2.1. Demographic Variables

The following characteristics were retrospectively collected: gestational age (weeks), birth weight (grams), gender, small for gestational age <p2.3 (SGA), administration of antenatal steroids, premature prolonged rupture of membranes (>24 hours, PPROM), Apgar scores, and surfactant therapy.

#### 2.2.2. Ventilator Settings

The charts of all preterm infants born after a pregnancy of ≤32 weeks and/or a birth weight ≤1500 g receiving hydrocortisone treatment were systematically reviewed for ventilation dependency. It was clear that in several days before the start of hydrocortisone treatment, no positive changes were seen in ventilator settings.

Data on ventilator settings were recorded within an hour before the start of hydrocortisone therapy and again one day and two days later: inspiratory oxygen fraction (FiO_2_), mean airway pressure (MAP; cm H_2_O), and arterial PaCO_2 _(mm Hg). Duration of mechanical ventilation and oxygen requirements at postmenstrual age of 36 weeks were documented.

#### 2.2.3. Ventilation Policy

The targeted level of oxygen saturation and PaCO_2_ was 88–93% and 45–60 mm Hg, respectively.

#### 2.2.4. Hydrocortisone Treatment

Generally, hydrocortisone was started when the postnatal age was at least more than 7 days and the child was ventilator-dependent with increasing oxygen requirements (FiO_2_ more than 0.40), prolonged dependency of extra oxygen (FiO_2_ of more than 0.40) based on chronic lung disease as evidenced by repeated pulmonary X-rays. Echocardiography was performed in all patients to exclude a hemodynamically significant patent ductus arteriosus before considering hydrocortisone. Hydrocortisone was given at a starting dose of 5 mg/kg/day tapering off to 1.25 mg/kg/day over a 22-day period. Depending on the response to therapy, steroid treatment was either prolonged or repeated. Weaning from the ventilator occurred on the basis of clinical condition and blood gases. 

Postnatal age at start of treatment, number of days treated with hydrocortisone, and hydrocortisone dose (mg/kg mean weight; mean weight: weight at the end of the treatment plus weight at the start of treatment divided by two) over the treatment period were recorded.

#### 2.2.5. Neurological Outcome

Infants were evaluated with a standardized neurologic examination at corrected ages of 0, 6, 15, and 24 months using items from the methods of Amiel-Tison and Grenier [14], Touwen [15], and a Griffiths' assessment [16]. Each child was examined for the presence of cerebral palsy. If present, neuromotor abnormalities including tone abnormalities/presence and type of cerebral palsy were recorded. Cerebral palsy was defined as a nonprogressive central nervous system disorder characterized by abnormal muscle tone in at least one extremity and abnormal control of movement and posture [8, 17].

### 2.3. Study Endpoints

Primary outcome measures were ventilator settings after day 1 and day 2 of treatment, duration of mechanical ventilation, successful extubation after start of hydrocortisone, CLD at postmenstrual age of 36 weeks, mortality, and development of cerebral palsy.

### 2.4. Statistical Analysis

Data are summarized as means ± SD or median and ranges. Data on respiratory function of day 0, day 1, and day 2, that is, FiO2, MAP, and PaCO_2_ were pairwise compared using Wilcoxon rank sum. A *P*-value < 0.05 was considered statistically significant. All analyses were performed using SPSS version 15.0.1 for windows (SPSS, Chicago, Il, USA).

## 3. Results

Fifty-five ventilation-dependent children fulfilled the inclusion criteria and were included in the present retrospective study.  Table 1 shows the most important patient characteristics whereas Table 2 shows respiratory characteristics.

**Figure 1:**
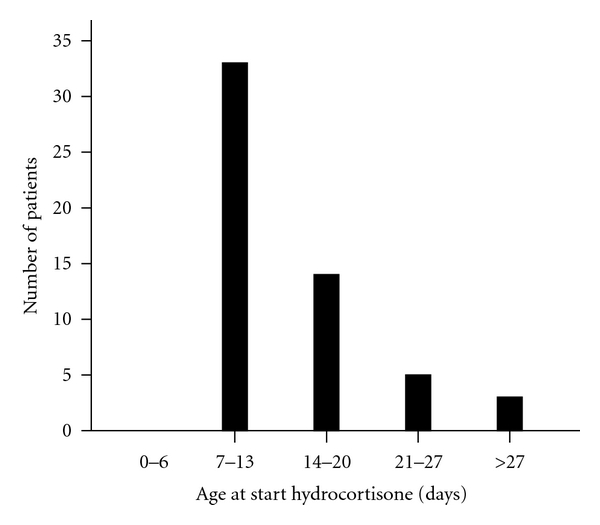
Age at start treatment with hydrocortisone.

Hydrocortisone was initiated at a median age of 11 days (Table 2 and Figure 1).

Median duration of hydrocortisone treatment was 21 days. In 52 out of 55 hydrocortisone-treated patients, the cumulative dose/kg mean weight could be calculated (mean weight: weight at the end of treatment plus weight at the start of treatment divided by two). Median cumulative hydrocortisone dose was 56.9 mg/kg mean weight.

### 3.1. Respiratory Effects

All 55 patients, who received hydrocortisone while being ventilated, could be successfully extubated at a median age of 4 days. In one case, it was hard to wean the child from the ventilator because of intercurrent periods of Staphylococcus epidermidis sepsis. This child was eventually weaned after 69 days.

**Figure 2:**
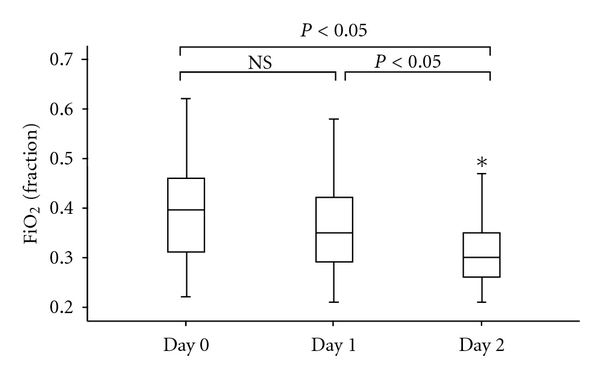
Values of oxygen requirement (inspirational oxygen fraction (FiO_2_)) on day 0 (baseline), day 1, and day 2 following treatment with hydrocortisone (HC). *P < 0.05 versus day 0 (baseline) and day 1. NS = non significant. FiO_2_ significantly decreases after day 2 compared with day 0 (baseline) and between day 1 and day 2.

Treatment with hydrocortisone was potent in reducing the need for extra oxygen (Figure 2). There was a significantly reduced requirement for oxygen after day 2 (*P*-value = 0.001) following start of hydrocortisone treatment and between day 1 and day 2 after start of hydrocortisone (*P*-value = 0.001). No significant reduced oxygen requirement was seen after one day (*P*-value = 0.006).

**Figure 3:**
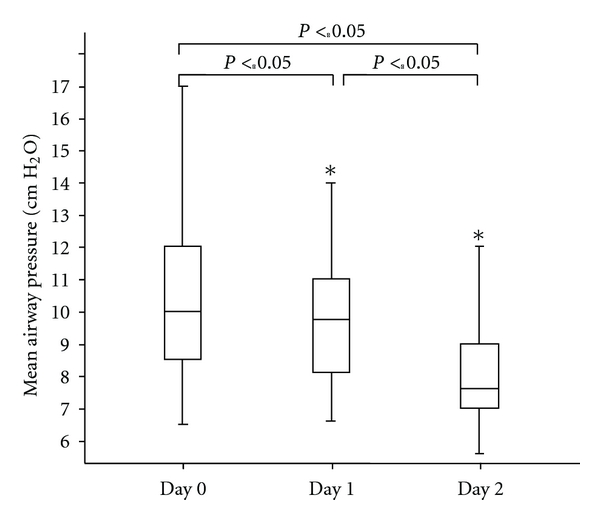
Values of Mean Airway Pressure (cm H_2_O) on day 0 (baseline), day 1, and day 2 following treatment with hydrocortisone (HC). *P < 0.05 versus day 0 (baseline) and day 1. Mean airway pressure significantly decreases on day 1 and day 2 compared with day 0 (baseline).

Maximal effect of hydrocortisone treatment on MAP was seen after day 1 after start treatment (Figure 3). This effect was significant with a *P*-value of 0.002. Furthermore, a significant effect was also seen after day 2 after start treatment (*P*-value< 0.001) and between day 1 and day 2 (*P*-value< 0.001).

**Figure 4:**
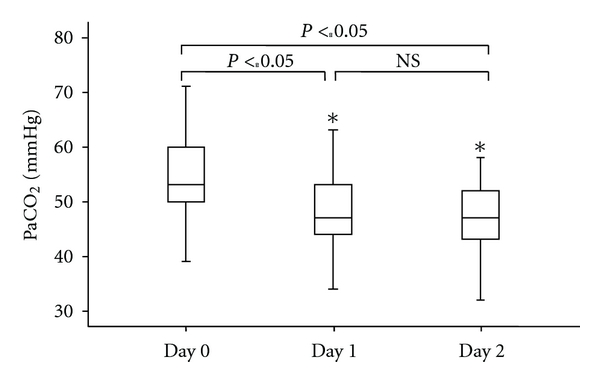
Values of PaCO_2 _(mmHg) on day 0 (baseline), day 1, and day 2 following treatment with hydrocortisone (HC). *P < 0.05 versus day 0 (baseline) and day 1. NS = non significant. PaCO_2_ significantly decreases on day 1 and day 2 compared with day 0 (baseline).

Hydrocortisone treatment had a maximal effect on PaCO_2_ one day after start treatment (*P*-value < 0.001) (Figure 4). No significant effect on PaCO_2_ was seen between day 1 and 2 after start hydrocortisone (*P*-value = 0.453).

After a gestational age of 36 weeks, 11 children (21.1%) required supplemental oxygen. Four children died before gestational age of 36 weeks, and in one surviving case supplemental oxygen requirement was not noted.

### 3.2. Neurodevelopmental Followup

Fifty-five ventilation-dependent children were included in this retrospective study. Of these, four were known to have died during neonatal period, leaving fifty-one patients available for followup at 24 months of corrected age (mean 24.1 ± 0.27 months). Standardized neurologic examination revealed that none of the 51 patients developed cerebral palsy.

## 4. Discussion

Our findings suggest that moderately early hydrocortisone therapy, in the dose mentioned, leads to a significant reduction in oxygen requirement (FiO_2_), PaCO_2_, and mean airway pressure in ventilator-dependent preterm infants more than 7 days old who were at risk for CLD.

Several studies indicate that low-dose dexamethasone administration after the first week of life significantly reduces the short-term pulmonary effects as failure to extubation and days of mechanical ventilation in ventilator-dependent preterm infants [5, 7, 18].

The study of Doyle et al. reported that dexamethasone (0.89 mg/kg/10 days) given at a median treatment age of 23 days to ventilator-dependent extremely low birthweight infants improved ventilator and oxygen requirements substantially and shortened the duration of intubation. There was little evidence for a reduction in either the mortality rate or the rate of oxygen dependency at 36 weeks [5].

Another study found that moderately early low-dose dexamethasone therapy for 7 days (tapering course, 0.2 to 0.1 mg/kg) had comparable beneficial effects to a high dose regimen. In both groups of infants (gestational age 24–30 weeks; birth weight 550–1290 g), FiO_2_ and MAP were significantly reduced [18].

A recent systematic review of 16 randomized, placebo-controlled trials (RTCs) including 1136 randomly assigned ventilated preterm infants more than 7 days of age assessed the effects of different cumulative dexamethasone doses on CLD and neurodevelopmental outcome. This paper concluded that higher cumulative dexamethasone doses (≥4 mg/kg) administered after the first week of life may decrease the relative risk for the combined outcome CLD or mortality. The risk of mortality or cerebral palsy was decreased by 6.2% for each incremental mg/kg cumulative dexamethasone dose when administered between 7–14 days of live. Furthermore, a significant reduction in short-term pulmonary effects as failure to extubation on day 3 and 7 and days of mechanical ventilation was seen in patients treated with dexamethasone after 7 days of live. These outcomes were independent of the cumulative dexamethasone dose [19].

Over the past years, concerns on the long-term neurodevelopmental consequences after dexamethasone therapy arose, when followup of a randomized controlled trial revealed a marked increase in neurodevelopmental dysfunction in neonates treated with dexamethasone compared with controls [11]. Since then, more reports of long-term negative neurodevelopmental sequelae have been published [10, 20, 21], and as a result The American Academy of Pediatrics stated in 2002 [22] that postnatal steroid administration, outside clinical trials, should be limited to “exceptional clinical circumstances”. In addition, a revised statement was issued by The American Academy of Pediatrics stating that current evidence is insufficient to make a recommendation regarding treatment with other glucocorticosteroids [23].

The long-term negative effects of dexamethasone necessitate a search for alternatives in the treatment of neonates with chronic lung disease. One of the possibilities frequently suggested in recent literature is the glucocorticosteroid hydrocortisone.

Few studies [12, 24–26] have investigated the use of hydrocortisone for treatment of CLD in premature infants and described long-term neurological outcomes, reviewed by Rademaker et al. [13].

Van der Heide-Jalving et al. retrospectively studied in a group of 25 preterm infants (mean gestational age 28.3 weeks; mean birth weight 1040 g) the effect of high-dose hydrocortisone on neurodevelopmental outcome. No significant differences were detected between the matched control group and hydrocortisone group with respect to neurological outcome, psychomotor development, and school performance at age 5–7 years. However, children treated with dexamethasone had a significantly less favourable school performance than their controls [12].

Another retrospective matched cohort study, Karemaker et al., compared behavioural outcome and motor skills in 192 preterm infants who neonatally either received dexamethasone or hydrocortisone for chronic lung disease. Dexamethasone- and hydrocortisone-treated groups were compared with a reference group and a group treated only antenatally with betamethasone. The groups were matched for gestational age, birth weight, gender, rate of respiratory distress syndrome, and rate of peri/intraventricular haemorrhage. At school age (7–10 years), the children treated with dexamethasone had a worse neurodevelopmental outcome than the reference group and betamethasone group, whereas the outcome of hydrocortisone-treated children did not differ from these two groups [24].

A large cohort study on long-term neurodevelopmental outcome in children following neonatal hydrocortisone treatment was reported in 2007 [25]. In this study, 226 preterm children (62 had been treated postnatally with hydrocortisone for CLD and 164 had not been treated with steroids) were followed up for 8 years. As this study was nonrandomized and treated children were generally sicker than untreated children, adjustments were made for gestational age, birth weight, gender, need for mechanical ventilation and small-for-gestational age. No differences were found in neurocognitive and motor performance between the two groups. Incidence of cerebral palsy and brain lesions on MRI was also similar.

Watterberg et al. reported that early low-dose hydrocortisone therapy for prophylaxis of early adrenal insufficiency to prevent bronchopulmonary dysplasia did not increase the incidence of cerebral palsy in extremely low birth weight infants. Treated infants had indicators of improved developmental outcome at 18 to 22 months' corrected age [26].

Recently, a prospective study was performed to evaluate the brain growth at term in preterm infants who did receive neonatal hydrocortisone for CLD. Thirty-eight preterm infants (*n* = 19 hydrocortisone, *n* = 19 controls) were matched for gestational age at birth. There were no differences in cerebral tissue volumes between the two groups at term equivalent age. In conclusion, no effect on brain growth was shown after treatment with hydrocortisone for CLD [27].

Our study supports the suggestion that hydrocortisone is not associated with cerebral palsy at 24 months adjusted age. According to findings of a recent study, we would have expected to find 2–5 cases of CP in our patients [28].

In 2010, Doyle et al. reviewed systematically the data from eight randomised controlled trials in newborn infants where hydrocortisone was compared with placebo to establish the balance between risks and benefits of hydrocortisone in newborn infants in the prevention or treatment of CLD [29]. In all eight trials, treatment was started in the first week of life; there were no trials of treatment started in infants who were chronically ventilated-dependent after the first week of life. A meta-analysis demonstrated little evidence to support the use of hydrocortisone (ranging in dose from 5 to 15 mg/kg) to prevent CLD; there were no improvements in important outcomes of mortality, or of rates in CLD or home oxygen dependence.

To our knowledge, no studies are performed that assess the short-term pulmonary effects following hydrocortisone treatment. Our study provides the first evidence that, besides dexamethasone, hydrocortisone also facilitates extubation, reduces oxygen and ventilator requirements. Since short-term pulmonary effects of dexamethasone and hydrocortisone are clinical comparable, and dexamethasone has proven long-term negative adverse effects, studies to use hydrocortisone in preterm ventilator-dependent infants at risk for CLD are warranted.

### 4.1. Limitations of the Study

The present study has also limitations. The primary limitation of our study is the lack of a control group. This lack is mainly related to its retrospective nature.

Furthermore, the data obtained in this study were limited by the fact that we performed a retrospective cohort study and not a randomized prospective study.

The sample size of our study population is small. The fifty-five patients in this study were born with a gestational age ≤32 weeks and/or birthweight of ≤1500 grams admitted over a period of five years to our NICU. Only a relatively small group fulfilled our inclusion criteria, a large group received hydrocortisone for other reasons than weaning from the ventilator.

Bearing in mind the above considerations, the results of this study still provides useful information.

We are convinced that this is the first step towards considering hydrocortisone as a therapy for prolonged ventilator-dependent children at risk for CLD. Finally, the results of the present study urge for a large double-blind randomized controlled trial on hydrocortisone versus placebo treatment for ventilator-dependent children at risk for CLD to clarify the discussion about which corticosteroid (dexamethasone or hydrocortisone) is safer for treatment of preterm infants with chronic lung injury.

## Figures and Tables

**Table 1:** Patient characteristics.

Characteristics	Total (*n* = 55)	%
Gestational age (wk)^a^	27.3 ± 1.4	
Birth weight (g)^a^	924 ± 231	
Gender^c^		
Female : male	17 : 38	
SGA (<p2.3)^c^	4	7.3
Antenatal steroids^c^	47	85.5
PROM > 24 h^c^	12	21.8
Apgar score^b^		
At 1 min	6 (1–10)	
At 5 min	8 (1–10)	
Mortality^c^	4	
Postnatal age at death (days)	13, 18, 22, 63	
Inotropes^c^	38	69.1
Patent ductus arteriosus^c^	31	56.4
Indometacin	30	96.8
Surgery	12 #	38.7
NEC^c^	2	3.6
NEC surgery requirement^c^	2	100
IVH^c^	21	38.2
Grades I-II	14	—
Grades III-IV	7	—
Cystic PVL^c^(grade II)	1	—

Data are shown as ^a^mean ± SD, ^b^median (range) or ^c^ *n*.

SGA: small for gestational age, PROM: premature rupture of membranes, NEC: necrotizing enterocolitis, IVH: intraventricular haemorrhage, and PVL: periventricular leukomalacia.

#In one case indometacin therapy was contraindicated because of IVH.

**Table 2:** Respiratory characteristics.

Characteristics	Total (*n* = 55)	%
Duration of ventilation (days)^b^	15.0 (5–69) *	
HFOV before treatment^c^	34.0	61.8
Postnatal age at start of HC (days)^b^	11 (7–44)	
Duration of HC (days)^d^	21.0 (9.0)	
HC dose (mg/kg mean weight)^d^	56.9 (28.2)	
Extubation after start HC (days)^d^	4.0 (4.0)	
IRDS^c^		
Grades I-II	23	41.8
Grades III-IV	32	58.2
Surfactant^c^		
No	6	10.9
Yes	49	89.1
Pneumothorax^c^	12	21.8

Data are shown as ^b^median (range), ^c^ *n* or ^d^median (interquartile range).

HFOV: high frequency oscillation ventilation; HC: hydrocortisone; IRDS: infant respiratory distress syndrome.

*In one case it took 69 days to wean the child from ventilator because of ongoing sepsis.
